# NIH Roadmap Interdisciplinary Research Initiatives

**DOI:** 10.1371/journal.pcbi.0010059

**Published:** 2005-11-25

**Authors:** Michael F Huerta, Gregory K Farber, Elizabeth L Wilder, Dushanka V Kleinman, Patricia A Grady, David A Schwartz, Lawrence A Tabak

A recent Perspective [1] suggested that the National Institutes of Health (NIH) Roadmap interdisciplinary research initiatives (http://nihroadmap.nih.gov/interdisciplinary/) embrace interdisciplinary teams without recognizing the importance of interdisciplinary individuals, and that these initiatives recognize something other than scientific questions as drivers of interdisciplinary research. As representatives of the implementation work group for these initiatives, we welcome this opportunity to clarify the approach that the interdisciplinary portion of the NIH Roadmap is taking to support science conducted beyond the borders of individual disciplines.

The NIH Roadmap interdisciplinary research work group issued seven requests for applications (RFAs). Recognizing that interdisciplinary individuals require knowledge of multiple disciplines, the work group emphasized individual training in four of these RFAs. Together, the training initiatives represent a coherent trans-NIH effort to not only provide training opportunities to individuals, but in the process, nurture and cultivate interest and investment in interdisciplinary research at the organizations to which those grants were awarded. The driving forces behind which disciplines are intertwined and drawn upon in these training activities are the questions that those participating are interested in pursuing (and which are, of course, relevant to the mission of the NIH).

Support provided under two other RFAs is also aimed at individuals—in these cases, to facilitate the development and enhancement of methods that work across disciplines contributing to behavioral, social, and biomedical research. Again, the scientific questions that interest those being funded will determine the nature of this interdisciplinary activity.

Finally, one RFA encouraged projects comprising teams of investigators from different disciplines to begin to develop new ways of thinking about, and addressing, significant research problems in research relevant to health and illness. Grants awarded under this RFA were specifically meant to facilitate thoughtful planning for what were hoped to become long-term interdisciplinary research projects. Again, these efforts are driven by the scientific questions that interest the participating investigators.

Very recently, the National Academies published “Facilitating Interdisciplinary Research” [2], a report that considered several aspects of this topic from many perspectives. We are delighted that the conceptualization and implementation of the NIH Roadmap interdisciplinary research initiatives conform well with the findings and recommendations presented in that report, including the importance of both individuals and teams in conducting such research.

It is clear that interdisciplinary research will play an increasingly significant role in improving human health. In recognition of this, the NIH is proceeding deliberately and rapidly to enhance its ability to advance this important and exciting paradigm. The Roadmap initiatives described above, which support both individual and team efforts and which are driven by the scientific questions that are of greatest interest to investigators and trainees, represent a major thrust in this direction.

## Abbreviations

NIH: National Institutes of Health
RFA: requests for application
